# Neural Substrates of Word Generation during Stroke Recovery: The Influence of Cortical Hypoperfusion

**DOI:** 10.1155/2007/430402

**Published:** 2007-02-12

**Authors:** V. Prabhakaran, S. P. Raman, M. R. Grunwald, A. Mahadevia, N. Hussain, H. Lu, P. C. M. Van zijl, A. E. Hillis

**Affiliations:** ^1^Johns Hopkins University School of MedicineBaltimoreMDUSA; ^2^Kennedy Krieger InstituteBaltimoreMDUSA

## Abstract

Several studies have demonstrated reorganization of cognitive and motor function caused by stroke. This study examined the influence of hypoperfused brain regions, in addition to the area of the infarct itself, on reorganization of the cognitive processes underlying word generation in stroke patients. In addition, we also sought to determine the influence of hypoperfusion on the blood oxygen level dependent/(BOLD) effect. Subjects with left and right subacute or chronic subcortical strokes, along with normal controls, were imaged while performing a verbal fluency task (word generation). The study population included six normal subject and six stroke patients with subcortical infarcts and cortical hypoperfusion in the middle cerebral artery territory who had recovered or improved markedly in word fluency. While normal subjects displayed a left-lateralized fronto-temporo-parietal and bilateral cingulo-striatal-thalamic-cerebellar network, the activation pattern of stroke patients was determined both by the hypoperfused regions and infarcted areas of the brain. Specifically, patients showed diminished BOLD effect in the cortical regions that were hypoperfused, even though their infarcts were subcortical, and showed increased BOLD effect in the homologous regions of the normal hemisphere. This finding raises the possibility that cortical hypoperfusion in the absence of infarct can cause shift of language functions to the opposite, intact hemisphere. However, reduced BOLD effect in the task relative to rest was found in hypoperfused regions in two patients, raising the possibility that regional function persisted, even though vascular reactivity was impaired. Results illustrate the complexities of functional imaging studies of recovery in patients with vascular lesions.

## 1. Introduction

Localization of cognitive processes to brain regions has mainly utilized the location of infarcted brain parenchyma in stroke patients or fMRI in normal subjects. Recent studies have used fMRI [45] or PET [3,22, 47,48] in recovered stroke patients to identify areas of the brain that can assume functions of the damaged part. Patients who have had strokes in the remote past have shown different levels of reorganization of brain networks, resulting in the ability to compensate for cognitive, perceptual, and sensori-motor deficits. Some have argued that this effect may be more pronounced for patients with left hemispheric infarcts, for whom the right hemisphere, hypothesized to be involved in "domain-independent" function, may be a valuable source of neural plasticity [22]. Others have reported shift of motor function to the intact hemisphere caused by cortical hypoperfusion, even without infarct [23,25,26]. FMRI allows the identification of functional networks that permit this reorganization. However, one difficulty in interpreting fMRI studies in stroke patients is that alterations in blood flow, blood volume, oxygen extraction, and metabolism may all contribute to alteration of the BOLD effect [30,46]. Several studies of patients who

ISSN 0953-4180/07/$17.00 © 2007 – IOS Press and the authors. All rights reserved have recovered motor function after stroke demonstrate that the BOLD effect in fMRI studies may be difficult to interpret in stroke patients who have areas of hypoperfusion or reduced vascular reactivity due to arterial stenosis [4,12,21,38,39]. Therefore, this study was undertaken to examine the influence of hypoperfused regions, in addition to the area of the infarct itself, on reorganization of *cognitive* processes and fMRI BOLD effect in patients with subacute or chronic subcortical infarcts and cortical hypoperfusion due to large vessel (middle cerebral artery) stenosis. We aimed to elucidate: 1) reorganization of the neural network underlying word generation caused by focal hypoperfusion and 2) the effects of hypoperfusion on the BOLD effect associated with language performance in patients with large vessel stenosis.

## 2. Materials and methods

### *2.1. Subjects*

The study sample consisted of six patients who had subcortical infarcts in the middle cerebral artery (MCA) distribution (ages 28–58, three males, three females), including four in the left MCA distribution and two in the right MCA distribution (Table 1). The time between the onset of the patient's stroke and the start of our imaging study varied between 6 months and 3 years. All patients were right handed and native English-language speakers. The four patients with left MCA infarcts had been aphasic at the time of the acute stroke, with various aphasic syndromes. All aphasic patients showed either complete recovery (three patients) or marked improvement from severe to mild deficits in language (including word fluency) at the time of the study (one patient). The two patients with right subcortical stroke and cortical hypoperfusion had diminished spatial attention and diminished word fluency at the time of the acute stroke, but showed normal performance on tests of both functions at the time of the current study. Six subjects (ages 24–57) with no known current or previous neurological disease, vascular disorders, structural brain alterations, or blood pressure medications were included as the control group. All subjects were right handed and native English-language speakers. The study was approved by the Institutional Review Board of Johns Hopkins Medical Institutions. All subjects provided written informed consent prior to their participation in the study.

### *2.2. Verbal fluency task*

The verbal fluency (word generation) task was first practiced outside the scanner, and then performed by all subjects while in the scanner. The task was a modified version of the verbal fluency paradigm that has been successfully applied in other studies with both normal and stroke patients [16,17]. This task was chosen because it has previously been shown to reliably result in widespread activation in the MCA territory in both hemispheres in normal subjects [10,42]. The task is divided into 11 epochs, with rest and activation conditions alternating – resulting in five activation epochs and six rest epochs. Each activation epoch lasted 60 seconds, whereas the intervening rest periods lasted 30 seconds. During the activation condition, the subjects were asked to generate words beginning with a letter that was presented to them on a screen located directly above the participant's head. Patients were asked to name words aloud, but subvocally (i.e., without moving their jaws, in order to reduce resultant motion artifact – a method first practiced and mastered outside the scanner). Verbal instructions were presented in the interval between the end of scan acquisition and the start of the subsequent TR-interval. All subjects demonstrated clear understanding of the instructions during practice and during scanning. Words were generated as quickly as possible by the subjects within each task epoch. At the end of the study (11 epochs), patients underwent a debriefing session in which they were asked to generate words beginning with the same letters that were presented in the scanner, and their answers were recorded as a behavioral measure, in order to assess compliance with the task while in the scanner. The aforementioned experimental protocol to evaluate compliance has been used in other comparable studies [1,9,10,20,32,42].

### *2.3. MRI scanning procedure*

Imaging was performed on a 1.5 Tesla whole-body MRI scanner (Philips Medical Systems, Best, the Netherlands). A body coil was used for RF transmitting and a quadrature head coil for receiving. Presentation of the visual instructions and letters to be used for the verbal fluency task to patients while in the scanner bed was done through the back-projection of images, via a video projector (Epson, Long Beach, CA), onto a screen located directly in front of the participant's head. Thirty contiguous 4 mm thick slices (no gap) were acquired parallel to the anterior commissure (AC) – posterior commissure (PC) plane. Other imag-

Table 1
Perfusion abnormalities and BOLD effect in ROIs. Highlighted blocks are abnormal

| ROI* | Normal subjects | SB (left stroke) | MS (left stroke) | MZ (left stroke) | HS (left stroke) | TG (right stroke) | JH (right stroke) |
|---|---|---|---|---|---|---|---|
| **LEFT** | | | | | | | |
| BA 6, 9/46 | Normal | **Hypoperfused** | Normal | Normal | Normal | Normal | Normal |
| | + BOLD | **No BOLD** | + BOLD | + BOLD | + BOLD | + BOLD | + BOLD |
| BA 37 | Normal | Normal | **Hypoperfused** | **Hypoperfused** | Normal | Normal | Normal |
| | + BOLD | + BOLD | **No BOLD** | **No BOLD** | + BOLD | + BOLD | **No BOLD** |
| BA 40 | Normal | **Hypoperfused** | **Hypoperfused** | Normal | Normal | Normal | Normal |
| | + BOLD | **No BOLD** | **No BOLD** | + BOLD | + BOLD | + BOLD | + BOLD |
| BA 44/45 | Normal | Normal | Normal | Normal | Normal | Normal | Normal |
| | + BOLD | + BOLD | + BOLD | + BOLD | + BOLD | + BOLD | + BOLD |
| BA 21 | Normal | Normal | Normal | Normal | Normal | Normal | Normal |
| | No BOLD | No BOLD | No BOLD | No BOLD | No BOLD | No BOLD | No BOLD |
| BA 3 | Normal | Normal | Normal | Normal | Normal | Normal | Normal |
| | No BOLD | No BOLD | No BOLD | No BOLD | No BOLD | + **BOLD** | No BOLD |
| **RIGHT** | | | | | | | |
| BA 6, 9/46 | Normal | Normal | Normal | Normal | Normal | **Hypoperfused** | Normal |
| | No BOLD | + **BOLD** | No BOLD | No BOLD | No BOLD | No BOLD | No BOLD |
| BA 37 | No BOLD | Normal | Normal | Normal | Normal | **Hypoperfused** | Normal |
| | | No BOLD | + **BOLD** | + **BOLD** | No BOLD | No BOLD | No BOLD |
| BA 40 | Normal | Normal | Normal | Normal | Normal | **Hypoperfused** | **Hypoperfused** |
| | No BOLD | No BOLD | No BOLD | No BOLD | No BOLD | No BOLD | **No BOLD** |
| BA 44/45 | Normal | Normal | Normal | Normal | Normal | **Hypoperfused** | Normal |
| | No BOLD | No BOLD | No BOLD | No BOLD | No BOLD | No BOLD | No BOLD |
| BA 21 | Normal | Normal | Normal | Normal | Normal | **Hypoperfused** | Normal |
| | + BOLD | + BOLD | + BOLD | + BOLD | + BOLD | **No BOLD** | No BOLD |
| BA 3 | Normal | Normal | Normal | Normal | Normal | **Hypoperfused** | Normal |
| | + BOLD | + BOLD | + BOLD | + BOLD | + BOLD | **No BOLD** | No BOLD |

*BA 37 – posterior, inferior temporal – involved in modality-independent lexical processing (e.g. naming and reading) [8,19,35,37]; BA 22 – Wernicke's area – involved in auditory word recognition [13,24,49]; BA 40 – supramarginal gyrus – involved in reading, writing, and phonology [36]; BA 44/45 – Broca's area – involved in speech articulation [17,27], written and spoken verb production [14,16], sentence formulation, syntax [2]; BA 9/46 – Dorsolateral prefrontal cortex involved in executive memory processes such as goal management, strategic retrieval, and reasoning [6,29,31,33,40,41]; BA 21 – involved in word and object meaning [44]; BA 3 – inferior post-central gyrus- sensory to face and mouth (engaged in speaking).

ing parameters for the BOLD experiment were: single-shot EPI, TR = 3000 ms, TE = 50 ms, FOV = 240 × 240 mm$^2$, matrix = 64 × 64, flip angle = 90°. Each fMRI experiment lasted for approximately 8 minutes.

The MRI data were reconstructed on the scanner and were transferred to a SUN Enterprise Server (SUN Microsystems, Mountain View, CA) for offline processing. An automated registration algorithm (AIR 3.0) was employed for motion correction. SPM 99b (Welcome Department of Cognitive Neurology, London, UK) was used to normalize the images to a standard space using an eight parameter liner transformation. The images were then spatially smoothed using a 8.0 mm Gaussian kernel. Finally, the statistical package contained within SPM 99b was used to estimate the effects of condition at each voxel according to the general linear model. The analysis was entered into SPM 99b as an epoch design of the fixed response/box car form, reflecting the eleven epochs of the study design and the appropriate activation and rest periods within the total scanning period. Images were corrected for differences in temporal acquisition between slices, realigned, motion-corrected, and normalized. Images were sinc interpolated to 2 × 2 × 2-mm$^3$ voxels and spatially smoothed with a Gaussian filter (7 mm full width half maximum). Images were high-pass filtered at 48 s to remove low-frequency drifts in blood oxygen level-dependent signal. No temporal derivative and no additional confounding variables were entered. Linear contrasts were used to test hypotheses about regionally specific effects, forming a statistical parametric map of voxel values for each contrast. These maps were transformed to the unit normal distribution and thresholded at $p = 0.001$ (uncorrected) to examine 10 regions of interest (five in each hemisphere). Distributional approximations from the theory of Gaussian fields were used to characterize the statistical significance of activation maps in terms of the probability that an observed peak height or region size could have occurred by chance across the entire imaged volume [34].

For five patients, perfusion images were obtained using bolus-tracking perfusion-weighted imaging (PWI). For these scans, we obtained single-shot gradient-echo EPI images (TR 2000 TE 60 ms with a 20 cc GdDTPA

bolus injected automatically at 5 cc/s followed by 15 cc normal saline at 5 cc/s. We used time-to-peak maps to determine the extent and site of hypoperfused brain. A region was considered hypoperfused if there was $>4$ second delay in time to peak arrival of contrast in that region, relative to the homologous region in the contralateral (normal) hemisphere [16,18]. This sequence generated 17 slices with 2 s temporal resolution. However, for one patient, due to technical reasons (lack of intravenous access), perfusion images were obtained using a continuous arterial spin labeling sequence (CASL) technique (transmitting/receiving head coil, TR $=$ 5000 ms, TE $=$ 42 ms, FOV $= 240 \times 240\ mm^2$, matrix $= 64 \times 64$, flip angle $= 90°$, duration 8 minutes 30 seconds) [7].

## 3. Results

### *3.1. Anatomic and perfusion imaging*

The location of the subacute/chronic infarct itself was ascertained with T2 weighted images at the time of this study. The area of infarct was confirmed by comparison with the areas of cellular depolarization (diffusion deficit) during the acute period. Of the four stroke patients with left MCA infarcts, all were subcortical in location with no involvement of cortex (patients MS, SB, MZ, HS). Similarly, of the two patients with right MCA distribution infarcts (patients TG and JH), both were located purely in the right basal ganglia, with no cortical involvement noted on T2-weighted or diffusion-weighted imaging. The major subcortical focus of the infarcts in the six stroke patients was the centrum semiovale and the basal ganglia.

Bolus-tracking PWI or CASL showed persistent hypoperfusion in all patients, despite substantial recovery of language or spatial attention. The patients with left subcortical infarcts showed larger areas of hypoperfusion, including frontal, parietal, and temporal cortex in the MCA vascular distribution, as well as subcortical parenchyma surrounding the region of the infarct. Similarly, of the two patients with right MCA distribution infarcts, one had cortical hypoperfusion encompassing the frontal, parietal, and temporal lobes (Patient TG), while the other had a region of hypoperfusion encompassing the entire right parietal lobe (Patient JH) (See Table 1). None of the anatomic or perfusion images were affected by significant artifact.

### *3.2. fMRI results*

Normal subjects. The verbal fluency task in normal control subjects yielded an activation pattern fairly consistent with other reports in the literature: fMRI imaging performed during epochs when the word generation task was performed yielded major cortical foci of activity in the (1) left posterior middle frontal gyrus (BA 6 and 9/46), (2) left posterior inferior frontal gyrus (BA 44/45); (3) left supramarginal gyrus (BA 40), and (4) left posterior inferior temporal gyrus (BA 37); right post-central gyrus (BA 3); and right middle temporal lobe (BA 21). Therefore, we examined these six areas in each hemisphere for activation in the stroke patients. There was also a consistent subcortical network recruited during the task, encompassing the cingulate gyrus, basal ganglia, thalamus, and cerebellum, all bilaterally. There was no hemispheric dominance noted in the subcortical activation pattern. During the "rest" epochs, the 30-second time periods in between the performance of the word generation task (during which the word "rest" was presented), normal subjects showed bilateral activation of the parieto-occipital cortex. The fMRI results yielded by the six control subjects during the task were used to create a composite image (See Fig. 1).

FMRI imaging in the six chronic stroke patients was dependent on the location of the subcortical infarct and the area of hypoperfusion as demonstrated on bolus-tracking perfusion or CASL imaging. Two patients (Patients HS and JH) showed no hypoperfusion in the regions of interest, and showed a normal cortical activation pattern, with diminished BOLD effect in the region surrounding the infarct in the left basal ganglia and centrum semiovale. The other patients demonstrated diminished activity in an area far greater than just the area surrounding the infarct itself, involving one or more of the regions of interest. In three patients with left subcortical infarcts, there was reduced BOLD effect, relative to normal controls, in left frontal, parietal, or temporal areas corresponding to the regions of hypoperfusion (Table 1). In patients with hypoperfusion involving left MCA territory, greater recruitment of right-hemisphere regions and less BOLD signal in the left fronto-temporo-parietal network compared to normals was observed, even though there was no infarct in the left fronto-temporo-parietal network. Similarly, TG, who showed hypoperfusion of right fronto-temporo-parietal regions, showed less BOLD signal than normal subjects in these areas. JH, who showed only right parietal hypoperfusion, showed the normal

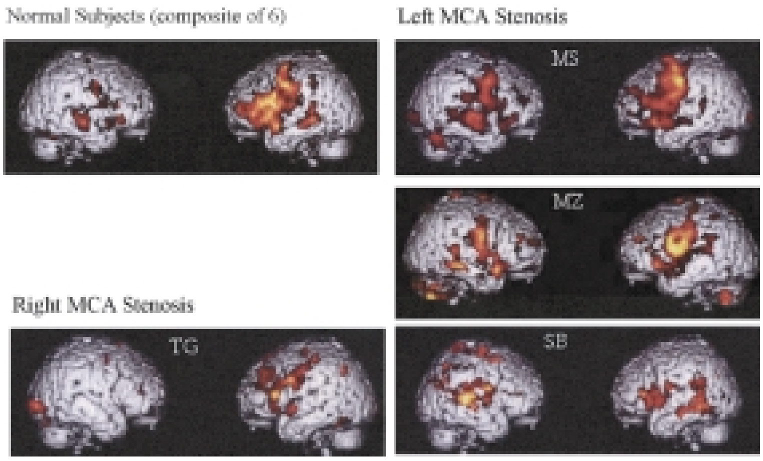

Fig. 1. BOLD effect for task versus rest. Top left panel. Composite results of fMRI of normal subjects. Bottom left panel. fMRI results for the one patient with hypoperfusion of regions of the right hemisphere that showed activation in normal subjects. Right panel. Individual fMRI results for the patients with hypoperfusion of regions of the left hemisphere that showed activation in normal subjects.

pattern, since the normal activation pattern does not encompass his region of hypoperfusion (right BA 40 and 37). The diminished BOLD signal in the region of hypoperfusion could be due to poor neural activation (e.g. due to lost white matter connections or impaired gray matter function) or reduced hemodynamic response to normal neural activation.

On the other hand, patient TG, who had a right subcortical stroke and hypoperfusion in the entire MCA distribution, displayed more BOLD signal in the right frontal, temporal, and parietal regions during rest (compared to the FAS task epochs); i.e., he showed a negative BOLD effect during the task (Fig. 2).

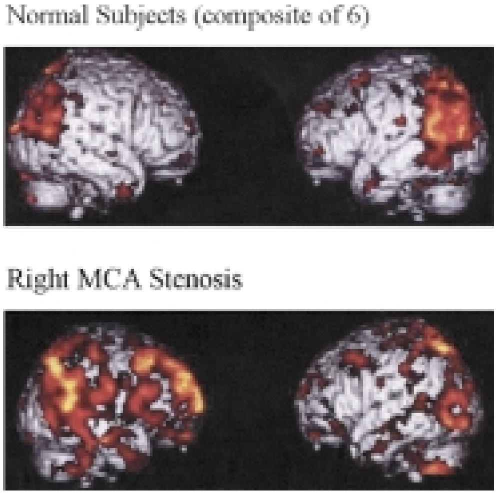

Fig. 2. BOLD effect for rest versus task. Top panel. Composite results of fMRI of normal subjects. Bottom panel. fMRI results for the one patient with hypoperfusion of regions of the right hemisphere that showed a negative BOLD response in the hypoperfused regions.

## 4. Discussion

Localization of cognitive functions on the basis of deficits associated with chronic strokes has generally proceeded on the assumption that there is a clear relationship between the site and extent of infarcted tissue and a patient's deficits. That is, it is assumed that if someone was cognitively intact prior to a stroke, but has impairment in some function following the stroke, the area that was damaged must be responsible for the function that became impaired with the stroke. However, several recent studies have shown that deficits may be better accounted for by the entire region of infarct and hypoperfused tissue [5,15,16,18,19]. In the past decade, localization of cognitive functions has relied largely on functional imaging studies, which are based on the assumption that task or event-associated increases in BOLD signal or regional cerebral blood flow in a given area indicates that that area of activation is important to the task or event. However, several recent studies combining functional imaging studies with transcranial magnetic stimulation to inhibit areas with increased signal have indicated that at least some areas that show reliable activation are not essential for the task or event that was associated with activation in that area [28]. Therefore, the relationship between task-associated signal change in an area and the role that area plays in the cognitive task or event, like the relationship between deficits and infarcts, may not be entirely straightforward.

In this study we evaluated the effects of tissue dysfunction (infarct or hypoperfusion) in areas that show fairly reliable activation during word fluency in functional imaging studies. In this study and previous studies, the fMRI signal pattern for the verbal fluency task was reasonably reliable across normal subjects (left lateralized network encompassing the inferior frontal lobe, superior temporal lobe, inferior parietal lobule, and bilateral cerebellum, basal ganglia, and thalamus). Our stroke patients showed fMRI signal dimunition (as compared to the normal pattern of signal activation) in frontal, temporal, or parietal cortical regions that were hypoperfused but not infarcted.

There are at least two non-exclusive accounts of the diminished BOLD signal in hypoperfused regions during task compared to rest. First, the reduced signal may be explained by reduced neural activation in this area (caused directly by hypoperfusion or caused by diaschisis that leads to hypoperfusion or reduced white matter connectivity). This reduced neural activation might cause a shift in function to the opposite hemisphere [23,25,26]. Alternatively (or additionally) the diminished BOLD effect could be due to reduced hemodynamic response to normal neural activation [4,12,21,38,39], because the BOLD effect is a reflection of the effects of cerebral blood volume (CBV) and oxygen extraction fraction (OEF) [30,46]:

$$\begin{aligned} \text{OEF} &= \text{oxygen consumption/oxygen delivery} \\ &= \text{CMRO2}/(\text{CBF*Ca}) \end{aligned}$$

In which CMRO2 = the cerebral metabolic rate of oxygen, CBF the blood flow, and Ca the arterial oxygen content. The BOLD effect is inversely proportional to OEF. Thus, a lack of vascular compliance but remaining activity (CMRO2) would result in a reduction of the BOLD effect or, in the ultimate absence of reactivity in a negative BOLD effect.

These two accounts may each best explain results of a subset of patients. The first account, reduced neural activation, can best account for results of the three patients with left frontal or temporal hypoperfusion, who each showed increased activation in homologous regions (BA6 and BA37) of the right hemisphere. This enhanced right hemisphere BOLD effect plausibly reflects reorganization of structure/function relationships, such that right hemisphere homologues assumed function of the left hemisphere in persistently hypoperfused regions. However, another explanation of the increased right hemisphere activation in patients compared to controls is that the task was simply more difficult for patients than for controls. In this case, the enhanced right hemisphere activation might have reflected increased effort or recruitment of additional neural or cognitive resources to complete the task, or even increased emotional response to the task.

The second account, reduced hemodynamic response to neural activation, may better explain results of TG. Patient TG, who showed a right subcortical infarct and hypoperfusion of the entire right frontoparietal and temporal cortex, showed a significantly negative BOLD signal in the right cortex during the word fluency task epochsis). These results might reflect increased OEF in response to neural activation during the task (compared to rest) in the absence of increased blood flow, resulting in negative BOLD effect during rest. The absence of a hemodynamic response can be explained by the patient's severe intracranial stenosis or by the positioning of an infarct in the white matter connections to the cortex. However, we did not assess the patient's vascular reactivity or focal oxygen extraction fraction, which might have provided better evidence that they did not have a normal hemodynamic response to neural activation [25,26]. An alternative explanation is that the patient was not simply resting during the rest epochs, but actively engaged in some cognitive task that recruited right frontotemporoparietal regions for the task during those epochs. However, since the same cognitive process must have been invoked by the subject without external stimuli over multiple epochs in cyclic fashion during the rest epoch, this is a less likely possibility.

Although we identified reduced fMRI signal changes in areas that were not infarcted, it was not the case that the patients' infarcts were irrelevant to fMRI signal change. All six patients had unilateral subcortical infarcts and lateralized subcortical hypoperfusion, but showed diminished subcortical fMRI signal bilaterally. Subcortical regions in both hemispheres seem to have been almost identically affected by a subcortical infarct localized only to one hemisphere. These observations raise the possibility that unilateral lesions in the basal ganglia or thalamus can diminish activity/fMRI signal in the homologous subcortical areas in the contralateral hemisphere.

In summary, we have demonstrated a complex relationship between subcortical infarcts, cortical hypoperfusion, and changes in fMRI BOLD signal associated with word fluency (a cognitive task that normally recruits widespread, left greater than right, frontotemporoparietal cortex). Our findings raise more questions than they answer, but extend to the cognitive domain two important observations from functional imaging of motor functions after stroke. First, in some cases, cortical hypoperfusion, even without infarct, may be sufficient to cause shift of cognitive function to homologous regions of the contralateral hemisphere (a result that has been reported in the motor domain [23]). Second, hypoperfusion due to arterial stenosis, at least with poor vascular reactivity, might preclude or reduce a BOLD response (or even cause a negative BOLD response) to neural activation in the hypoperfused area [4,12,21,38, 39]. A recent study has shown that in stroke patients studied with both PET and MRI within 24 hours, there are areas of low regional cerebral blood flow (rCBF) with high oxygen extraction fraction (OEF), areas of low CBF with low or variable OEF, and areas of high rCBF with low OEF both within and beyond abnormalities on DWI [11]. Therefore, at least in the domains of language and cognition, confirmation of these accounts of reduced fMRI signal in undamaged cortex normally involved in the task being studied requires further exploration of the relationship between the focal BOLD response, oxygen extraction, neural activation, and task performance with multimodality imaging.

## Acknowledgements

The research reported in this paper was supported by NIH R01: NS047691 and P41 RR15241 and DC05375.
